# Intracavitary vaginal brachytherapy using a custom balloon applicator

**DOI:** 10.1002/jmrs.235

**Published:** 2017-06-29

**Authors:** Dean B. Paterson, Shelley M. Pearson, Andrew N. Wilson

**Affiliations:** ^1^ Radiation Treatment Department Blood and Cancer Centre Wellington Regional Hospital Wellington New Zealand

**Keywords:** Brachytherapy, custom applicator, intracavitary brachytherapy, vaginal cancer

## Abstract

A custom balloon applicator was created to deliver intracavitary high‐dose‐rate brachytherapy to a patient with a superficial vaginal cancer. The patient was unable to be treated conventionally due to an extremely narrow introitus that prevented the introduction of a conventional cylinder applicator. The custom applicator was constructed by inserting a straight titanium tandem applicator through the drainage lumen of a Foley catheter. The applicator was inserted and the catheter balloon was inflated when positioned at the vaginal apex. Three brachytherapy treatments were performed using this technique. Individual balloon eccentricities resulted in small radial tandem offsets within the balloon. This phenomenon was exploited by orientating the tandem offset in the direction of the target volume. Acceptable dosimetry was achieved for all fractions and the procedure was very well tolerated. The custom applicator was a viable solution that was safely developed in a short time frame using equipment readily available in our department.

## Introduction

Intracavitary vaginal brachytherapy is a common adjuvant treatment for endometrial carcinoma and superficial vaginal carcinoma.^1^, ^2^ Intracavitary brachytherapy is suitable for vaginal carcinomas invading a depth up to 0.5 cm whereas bulkier disease should be treated with interstitial brachytherapy.^2^ To avoid under‐dosing tumour, it is imperative that the applicator conforms to the vaginal mucosa without air gaps.^1^ Cylinders and ovoids, with standardised lengths and diameters, are commonly used. Cylinders are able to treat a greater length; however ovoids may achieve better mucosal contact in the presence of a widened vaginal apex.^1^ Multi‐channel cylinders offer a greater level of dosimetric control.^1^ Custom applicators are required when standard applicators do not conform or adapt to patient specific anatomy. For patients with a narrow introitus, standard applicators wide enough to achieve conformity at the vaginal apex may be impossible to insert or can cause significant pain on insertion. This article describes a custom intracavitary balloon applicator designed to deliver high‐dose‐rate (HDR) brachytherapy to a patient with a superficial vaginal adenocarcinoma. The applicator design, planning process, treatment process and resultant dose distribution are reported.

## Materials and Methods

### Clinical case

A 75‐year‐old female with a superficial T1 vaginal adenocarcinoma was referred to our institution for chemo‐radiation using external beam radiation therapy (EBRT) and HDR brachytherapy. The patient had a hysterectomy many years earlier due to fibroids. During work‐up, clinical examination found that the hymen was intact and only a fingertip could be inserted past the introitus. At examination under anaesthesia an incision was made to open the introitus, however, optimal visual examination could only be achieved using a cystoscope. A 1.3 cm exophytic lesion was identified on the left lateral vaginal wall, 1–1.5 cm caudal to the vaginal apex. It was noted that the vaginal apex was far more capacious than the introitus. Pelvis magnetic resonance imaging (MRI) showed no evidence of paravaginal or nodal disease. The vaginal length (apex to introitus) was estimated at 8 cm.

A course of EBRT to the whole pelvis (45 Gy in 25 fractions, five fractions per week) with vaginal brachytherapy (24 Gy in four fractions, two fractions per week) interdigitated during the final 2 weeks was prescribed. Concomitant weekly Cisplatin was administered. Brachytherapy was prescribed at a depth of 0.5 cm along a 5 cm treatment length from the vaginal apex. A vaginal cylinder narrow enough (diameter <2 cm) to insert through the patient's introitus would not conform to the vaginal apex. Therefore, a custom brachytherapy applicator was pursued. The patient provided signed consent to having her case published.

### Applicator construction

**Figure 1:**
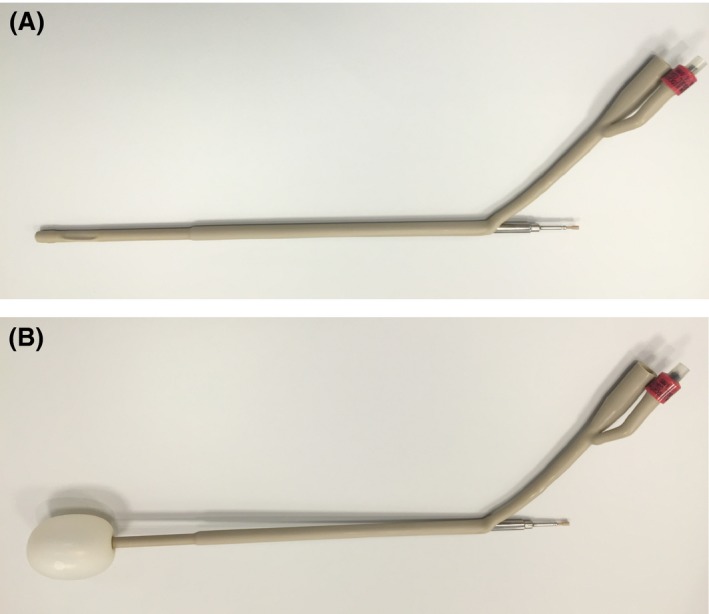
(A) Un‐inflated custom balloon applicator. Constructed by inserting a straight titanium tandem applicator into the drainage channel of an 18G Foley catheter. (B) Custom balloon applicator inflated with 45 mL saline solution. The tip of the Foley catheter has been removed from the top of the balloon.

A straight titanium tandem applicator (Mick Radio‐Nuclear Instruments, Mount Vernon, NY) was inserted through an 18‐gauge Foley catheter balloon (C. R. Bard Inc, Murray Hill, NJ) (Fig. 1A). The catheter tip was cut from the top of the balloon and an incision was made in the drainage lumen of the catheter. The tandem was inserted so the tip of the tandem was adjacent with the top of the balloon. A thin rubber sleeve (applicator cleaning cap) was placed on the tandem tip prior to insertion to prevent it slipping inside the balloon. The balloon was deflated to expel any existing air. Forty‐five millilitres (45 mL) of saline was then inserted through the balloon portal, inflating the balloon to the treatment volume, to allow evaluation of the size, shape and integrity of the balloon (Fig. 1B). A new applicator was constructed for each insertion.

### Insertion procedure

The patient was placed in the dorsal lithotomy position. No anaesthesia or sedation was required during the procedure. The patient's bladder was catheterised. The applicator balloon was completely deflated and inserted to the vaginal apex. Where there was a radial offset of the tandem within the balloon, due to individual balloon eccentricities, the applicator was orientated so that the offset was towards the patients left (tumour location). The rotational orientation of the applicator was marked on the tandem and the balloon was inflated with 45 mL of a 5% radiographic contrast and saline solution. Trans‐abdominal ultrasound was used to verify inflation. A small amount of vaginal packing was used to stabilise the applicator, which remained firmly immobilised without the aid of a clamp. The patient's legs were lowered onto a knee rest which also supported the tandem and reference marks for the applicator position were drawn on the patient's thighs.

### Imaging

Prior to the first treatment insertion, computed tomography (CT) (Phillips Healthcare, Amsterdam, the Netherlands) and T2‐weighted MRI (Philips Healthcare) imaging was performed with the applicator in situ. The CT (0.15 cm slice thickness) was used to determine the feasibility of the technique. MRI was used to evaluate tumour response to EBRT and for delineation of a high‐risk clinical target volume (HRCTV) using the GEC‐ESTRO formalism. Subsequent CT‐imaging was performed after each treatment insertion. Prior to imaging, 60 mL of fluid was introduced to the patient's bladder. Each CT was assessed to ensure the balloon conformed to the vaginal mucosa without air gaps. Treatment planning was performed on the acquired CT data set for each insertion.

### Treatment planning

**Figure 2:**
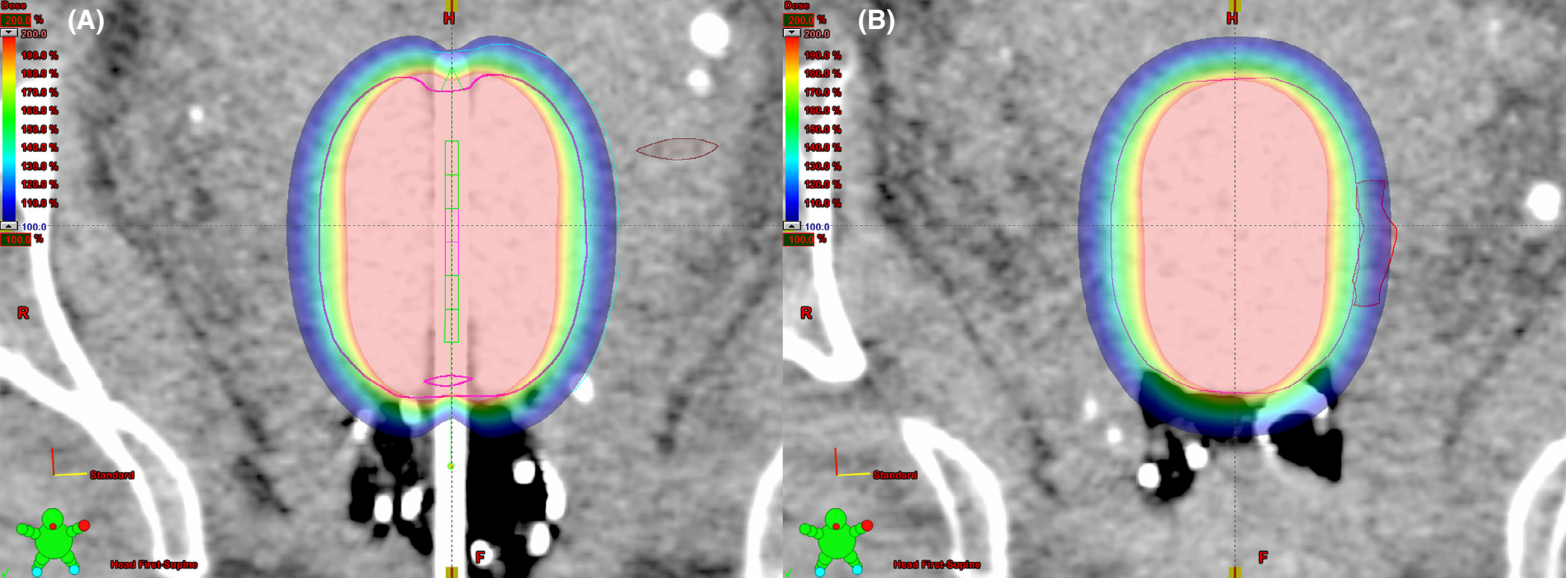
(A) Para‐coronal CT simulation image aligned to the tandem showing isodose distribution obtained on fraction one. The inflated balloon is contoured in magenta and the 5 cm long reference line is displayed as a dashed line 0.5 cm deep to the balloon on the patient's left. The colour wash is set to display 100–200% of the prescribed dose. (B) As above with viewing planes aligned through the HRCTV (red).

Treatment planning was performed using BrachyVision v11.0 (Varian Medical Systems, Palo Alto, CA). Each plan was optimised to deliver 100% of the prescribed dose to a 5 cm long reference line, placed 0.5 cm from the balloon surface on the patient's left (tumour location) (Fig. 2). The 200% isodose was mostly contained within the applicator. The HRCTV was transferred to planning CT scans by registering the images according to the applicator. Bladder, rectum and sigmoid were contoured on the CT for dose reporting.

The maximum diameter and length of the balloon were measured for each fraction using the planning software. The anterior‐posterior and lateral radial offset of the tandem from the centre of the balloon were also measured.

### Treatment delivery

Treatment was delivered using a Varisource iX afterloader (Varian Medical Systems). Prior to treatment 60 mL of fluid was introduced to the patient's bladder. Trans‐abdominal ultrasound was used to verify balloon inflation. Markings on the patient's thighs were used to visually confirm the orientation and position of the tandem. After treatment the balloon was deflated and the applicator removed. Analgesia was required for applicator removal for the final fraction only.

## Results

The patient received 36 Gy in 20 fractions of EBRT, with five cycles of chemotherapy. EBRT was terminated early as the patient developed severe bladder toxicity. The residual tumour was visible on the pre‐brachytherapy MRI and was well encompassed by the 100% isodose. The decision was made to proceed with brachytherapy, however, the prescription was reduced to 18 Gy in 3 fractions (two fractions per week). During EBRT the patient experienced mild to moderate bowel toxicity. These changes appeared to be associated with long‐standing diverticulosis.

**Figure 3:**
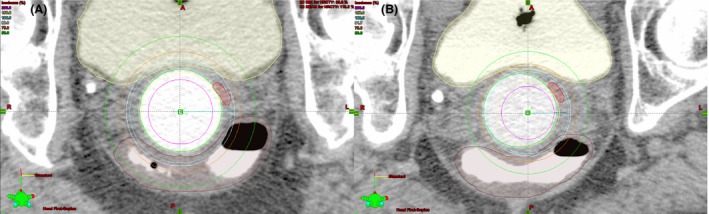
(A) Para‐transverse view of Isodose distributions for fraction one with a central tandem. (B) Fraction two with a tandem offset 3.5 mm to the patient's left. Yellow: bladder; brown: rectum; red: HRCTV; green: balloon.

Balloon dimensions were consistent with an inter‐fraction deviation of 0.1 cm for length and 0.2 cm for diameter (Table 1). The balloon length (4.8–4.9 cm) was adequate for a treatment length of 5 cm. The tandem was central in the balloon for fraction one. Radial offsets of the tandem position within the balloon, were present for fractions two and three. The offsets were due to individual balloon eccentricities which were visually obvious during applicator construction. Rather than discarding these balloons, this phenomenon was exploited. For fractions two and three, the known radial offset was intentionally positioned towards the known tumour location (Fig. 3). This resulted in lower dose to the rectum 2 cc and uninvolved vaginal mucosa compared to fraction one whilst the HRCTV D90 remained consistent (Table 2). The anterior tandem offset (2.5 mm) present for fraction three corresponds with the highest bladder 2 cc dose (100.7%).

**Table 1:** Custom balloon applicator technical parameters measured from CT simulation images

	Balloon diameter (mm)	Balloon length (mm)	Anterior‐posterior tandem offset^a^ (mm)	Lateral tandem offset^a^ (mm)
Fraction 1	40	48	0	0
Fraction 2	39	49	0	+3.5
Fraction 3	38	49	+2.5	+1.5

a Offset of tandem from balloon centre.

**Table 2:** Summary of dosimetric quantifiers for each brachytherapy plan and for the total treatment course

Dosimetric quantifiers	Fraction 1 dose (%)	Fraction 2 dose (%)	Fraction 3 dose (%)	Total course EQD2^a^ (Gy)
HRCTV^b^				
D90	104.9	108.3	108.4	61.8
D98	99.1	102.9	102.4	59.9
D100	95.6	98.7	99.1	58.7
Bladder				
D2 cc	95.3	70.2	100.7	61.5
D0.1 cc	112.0	85.7	121.5	71.0
Rectum				
D2 cc	103.5	84.5	79.50	61.6
D0.1 cc	119.3	109.7	96.8	72.0
Sigmoid				
D2 cc	36.7	49.7	31.5	42.3
D0.1 cc	48.2	72.3	40.2	46.9

a The total course dose values are based on the cumulative dose of EBRT and brachytherapy and are expressed in equivalent dose in 2 Gy fractions (EQD2) using an alpha‐beta value of 3 for organs at risk and an alpha‐beta value of 10 for tumour. It is assumed that all structures receive the full EBRT dose. The prescription dose for each brachytherapy plan was 6 Gy.

b High‐risk clinical target volume.

At 3 months follow‐up the patient was doing well. She had slight vaginal discharge and occasional urgency and nocturia. Her long‐standing variable bowel pattern remained. To visually assess tumour response another examination under anaesthetic would be required, it was deemed inappropriate to put the patient through that process again.

## Discussion

An acceptable plan would not have been achieved with any of the standard intracavitary applicators available in our department (single or multi‐channel cylinder and ovoids). The patient's introitus was too narrow for cylinder insertion and it would have been impossible to safely achieve a treatment length of 5 cm with ovoids. Interstitial brachytherapy was considered, however, the tumour size, superficial location and difficulties with visualisation meant this option was not pursued. The custom balloon applicator was a viable solution and could be safely developed in a short time frame using equipment readily available in our department.

A number of alternative applicators exist which may have been suitable for this case. Miller et al.^3^ reported on a series of patients treated for endometrial carcinoma with post‐operative vaginal brachytherapy using a balloon applicator. The Mammosite (Hologic Inc., Marlborough, MA), a single‐channel spherical balloon applicator designed for breast brachytherapy, was used to treat the proximal third of the vagina. The rationale for using a balloon applicator was to improve conformity with the vaginal mucosa compared with a standard cylinder. Optimal balloon distension was patient specific. The average balloon volume was 47 cc, which was very close to the 45 cc volume we used. Excellent target volume coverage and acceptable bladder and rectal doses were achieved, with no acute complications. The successful use of an intra‐vaginal balloon applicator supports the approach taken for our case.

The Capri (Varian Medical Systems) is a multi‐lumen balloon applicator, specifically designed for vaginal brachytherapy.^4^ It consists of a central channel surrounded by two concentric rings each containing six additional channels. The diameter of an un‐inflated Capri is 2.8 cm^4^ meaning it is unlikely this applicator would pass through the introitus of our patient. The commercially available balloon applicators discussed are not available in our department.

Wiebe et al.^5^ described a customised mould applicator developed as a solution for a patient with an irregular vault configuration characterised by a wide vaginal apex (4.4 cm) relative to introitus (2.5 cm). A 3D digital model was created from CT images with contrast soaked vaginal gauze in place. Patient‐specific catheter tracks were incorporated into the model which was then converted into a resin applicator using stereolithography. To reduce the cross‐sectional area during insertion, the applicator was designed in two sections so that one half could be inserted followed by the second half which was then attached to the first. At no point did the diameter exceed 3.0 cm at introitus. The applicator conformed well to the mucosa and resulted in significant dosimetric improvements compared to a standard cylinder. The resources required to produce this type of patient specific applicator were not available in our department. Other mould techniques were considered and discounted due to difficulties removing a mould.

The maximum bladder and rectum 2 cc doses are within the range reported for standard cylinder plans. Caon et al.^6^ conducted a retrospective analysis to assess organ at risk dose in 41 patients who underwent 3D planning for vaginal vault brachytherapy using a standard cylinder. Standardised plans were prescribed at a 0.5 cm depth to the proximal 4 cm of the vagina. In the Caon et al.^6^ series, the mean bladder 2 cc dose was 80% (14.3–124.3%) and the mean rectum 2 cc dose was 81.4% (22.9–140%) of the prescribed dose. In our case, bladder 2 cc dose ranged from 70.2% to 100.7%. This range is primarily due to differences in the radial offset of the tandem within the balloon. The highest bladder 2 cc dose was recorded for fraction three, where there was a 2.5 mm anterior tandem offset. The rectal 2 cc dose ranged from 79.5% to 103.5%. The highest rectum dose was recorded for fraction one where no radial offset of the tandem was observed. The prescribed treatment length for our case was 5 cm. It is likely that this would be associated with a higher rectum 2 cc dose than a 4 cm treatment length.

Limitations of our technique include the eccentric nature of the balloons which resulted in a radial offset of the tandem within the balloon. This could present an issue if a symmetrical dose distribution was an absolute requirement. We decided to take advantage of the offset, if present, rather than discard the balloon for one with no offset. This resulted in excellent HRCTV coverage and lower dose to the uninvolved vaginal mucosa and rectum. Another limitation is that the balloon length was limited to 5 cm. This was adequate to achieve a 5 cm treatment length however would be inadequate for distal vaginal lesions.

A number of technical issues were encountered. Some types of Foley catheter tested were unsuitable as the catheter tip could not be removed without compromising the integrity of the lumen within the balloon. Other catheter and balloon devices tested could not inflate to the required length or volume, while others could inflate far beyond the treatment volume but were not rigid enough to hold the tandem in place. A rubber sleeve was needed on the tip of the tandem to prevent it from slipping within the catheter channel. This worked well and the tandem position was very stable within the balloon.

## Conclusion

A custom balloon applicator was created to deliver intra‐vaginal brachytherapy to a patient with a very narrow introitus relative to the vaginal vault. The custom applicator was a viable and safe solution that resulted in an acceptable dose distribution and was well tolerated by the patient.

## Conflict of Interest

The authors declare no conflict of interest.

## References

[1] Small W , Beriwal S , Demanes DJ , et al. American Brachytherapy Society consensus guidelines for adjuvant vaginal cuff brachytherapy after hysterectomy. Brachytherapy 2012; 11: 58–67. 22265439 10.1016/j.brachy.2011.08.005

[2] Beriwal S , Demanes DJ , Erickson B , et al. American Brachytherapy Society consensus guidelines for interstitial brachytherapy for vaginal cancer. Brachytherapy 2012; 11: 68–75. 22265440 10.1016/j.brachy.2011.06.008

[3] Miller DA , Richardson S , Grigsby PW . A new method of anatomically conformal vaginal cuff HDR brachytherapy. Gynecol Oncol 2010; 116: 413–8. 19892389 10.1016/j.ygyno.2009.10.044

[4] Kuo H‐C , Mehta KJ , Yaparpalvi R , et al. Feasibility study and optimum loading pattern of a multi‐ring inflatable intravaginal applicator. J Contemp Brachytherapy 2013; 5: 93–100. 23878554 10.5114/jcb.2013.35580 PMC3708147

[5] Wiebe E , Easton H , Thomas G , Barbera L , D'Alimonte L , Ravi A . Customized vaginal vault brachytherapy with computed tomography imaging‐derived applicator prototyping. Brachytherapy 2015; 14: 380–4. 25630618 10.1016/j.brachy.2014.12.006

[6] Caon J , Holloway C , Dubash R , Yuen C , Aquino‐Parsons C . Evaluating adjacent organ radiation doses from postoperative intracavitary vaginal vault brachytherapy for endometrial cancer. Brachytherapy 2014; 13: 94–9. 24269147 10.1016/j.brachy.2013.09.007

